# Inhibition of *N*‐myristoyltransferase1 affects dengue virus replication

**DOI:** 10.1002/mbo3.831

**Published:** 2019-03-07

**Authors:** San Suwanmanee, Yuvadee Mahakhunkijcharoen, Sumate Ampawong, Pornsawan Leaungwutiwong, Dorothée Missé, Natthanej Luplertlop

**Affiliations:** ^1^ Department of Microbiology and Immunology Faculty of Tropical Medicine Mahidol University Bangkok Thailand; ^2^ Department of Tropical Pathology Faculty of Tropical Medicine Mahidol University Bangkok Thailand; ^3^ MIVEGEC UMR 224, Université de Montpellier, IRD, CNRS Montpellier France

**Keywords:** dengue virus, envelope protein, *N*‐myristoyltransferase1, viral replication

## Abstract

Dengue virus (DENV) causes dengue fever, a self‐limiting disease that could be fatal due to serious complications. No specific treatment is currently available and the preventative vaccine is only partially protective. To develop a potential drug target for dengue fever, we need to understand its biology and pathogenesis thoroughly. *N*‐myristoyltransferase (NMT) is an N‐terminal protein lipidation enzyme that catalyzes the covalent cotranslational attachment of fatty acids to the amino‐terminal glycine residue of a number of proteins, leading to the modulation of various signaling molecules. In this study, we investigated the interaction of dengue viral proteins with host NMT and its subsequent effect on DENV. Our bioinformatics, molecular docking, and far‐western blotting analyses demonstrated the interaction of viral envelope protein (E) with NMT. The gene expression of NMT was strongly elevated in a dependent manner during the viral replication phase in dendritic cells. Moreover, NMT gene silencing significantly inhibited DENV replication in dendritic cells. Further studies investigating the target cell types of other host factors are suggested.

## 1 INTRODUCTION

Dengue virus (DENV) infection is a neglected tropical disease that is widespread in tropical and subtropical regions. Around 390 million infections are reported annually, with 500,000 severe cases resulting in ~25,000 deaths per year (Horstick, Tozan, & Wilder‐Smith, 2015). Dengue fever is usually a self‐limiting disease, but some cases can result in severe, life‐threatening symptoms, such as dengue hemorrhagic fever, which causes bleeding that can lead to dengue shock syndrome, characterized by severe plasma leakage, prolonged shock, and multiple organ failure. Because the currently available vaccine is not completely effective and there is no specific treatment for dengue fever, treatment is supportive and includes fluid therapy. However, a fully effective preventative vaccine is under development (Rajapakse, Rodrigo, & Rajapakse, 2012; Shim, 2017).

The DENV genome encodes 10 viral proteins consisting of three structural proteins, namely capsid (C), premembrane/membrane (prM/M), and envelope (E) proteins; and seven nonstructural proteins—NS1, NS2A, NS2B, NS3, NS4A, NS4B, and NS5 (Rustin et al., 2007). For viral replication to occur, a host element is needed to produce the new virion. Consequently, it is very important that the viral‐host interaction be achieved during the viral life cycle (Noppakunmongkolchai et al., 2016). *N*‐myristoyltransferase (NMT) belongs to the GCN5 *N*‐acetyltransferase superfamily and is the key enzyme responsible for catalyzing the myristoylation process, which is the central switch of several cellular signaling pathways necessary for growth and cellular proliferation. Moreover, the fundamental role of NMT as being essential for cell survival makes it a potential drug target for the treatment of cancers, parasitic infections, and other infectious diseases. NMT has two isoforms, NMT1 and NMT2, encoded by different genes and with different specificities.

NMT1 has exhibited numerous dominant functions, such as in vivo inhibition of tumor growth in NMT1 knockdowns and defective myelopoiesis in NMT1 knockdown mouse embryos (Kumar, Singh, Dimmock, & Sharma, 2011; Kumar & Sharma, 2015; Zhao & Ma, 2014). A previous study has shown that the disruption of hNMT1 long form, but not human NMT2, could inhibit HIV‐1 replication in vivo (Takamune et al., 2008). Previous NMT1 studies have focused on cancer development, diagnostic biomarkers, and chemotherapeutic targets (Selvakumar et al., 2007; Shrivastav, Varma, Saxena, DeCoteau, & Sharma, 2007; Thinon, Morales‐Sanfrutos, Mann, & Tate, 2016). Several studies have investigated the biology and pathogenesis of NMT from malaria and leishmania within its potential as a novel drug target by exploiting the inhibition effect observed when NMT expression is disrupted (Banerjee, Arora, & Murty, 2012; Bell et al., 2012; Brannigan et al., 2010; Tate, Bell, Rackham, & Wright, 2014). Furthermore, recognition of NMT as a cell wall target of *Aspergillus fumigatus* infection has also identified NMT as a potential therapeutic drug target (Fang et al., 2015; Valiante, Macheleidt, Foge, & Brakhage, 2015), and NMT has been identified as a novel antifungal target of *Candida albicans* (Sikorski et al., 1997; Wiegand et al., 1992). A study of the role of NMT in relation to human immunodeficiency virus proteins has revealed that the myristoylation of Gag and Nef proteins is a key for viral replication and virulence (Seaton & Smith, 2008). Concerning *Flavivirus* studies, an in silico analysis predicted a myristoylation site (Maurer‐Stroh & Eisenhaber, 2014), while in silico studies focusing on the DENV‐E protein also predicted posttranslational modification sites and investigated their role in pathogenesis (Ruhul Amin, Mahbub, Sikder, & Karim, 2010).

Studies investigating *Flavivirus* NMT both in silico and in vitro are rare but are needed to develop therapeutic approaches based on the factors involved in viral‐host interactions. We hypothesized that NMT1 might play some role in *Flavivirus* infection. Thus, in this study, we performed both in silico and in vitro analysis to elucidate the role and effect of NMT under the conditions of *Flavivirus* infection. We used DENV as our study model, focusing on the biology and physiological relevance of host‐virus interactions.

## 2 MATERIALS AND METHODS

We investigated the interaction between host NMT and DENV, particularly concerning virus replication, using the DENV Serotype 2 strain 16681 with human dendritic cells.

### 2.1 Dendritic cell isolation and generation

The buffy coat was diluted 1:1 with sterile phosphate‐buffered saline (PBS), and peripheral blood mononuclear cell (PBMC) isolation was performed using Ficoll^®^‐Paque PREMIUM density gradient medium (GE Healthcare, Little Chalfont, UK) according to the manufacturer's recommendations. Isolated PBMCs were cultured in T75 flasks for monocyte selection and stimulated to monocyte‐derived dendritic cells with recombinant human IL‐4 and granulocyte‐macrophage colony‐stimulating factor (Luplertlop et al., 2006). An African green monkey kidney cell line (Vero) was kindly provided by Dr A. Thontiravong of the Faculty of Veterinary Medicine, Chulalongkorn University, Thailand. The cells were cultured in RPMI 1640 media (Gibco, Waltham, MA). Lilly Laboratories Cell—Monkey Kidney 2 (LLC‐MK2) cells were cultured in Dulbecco's modified Eagle's medium (Gibco). All cell cultures were supplemented with 10% fetal bovine serum and 1% penicillin/streptomycin (Gibco) and incubated at 37°C, 5% CO_2_ in a humidified incubator.

### 2.2 Virus, viral culture, and viral infection

DENV Serotype 2 strain 16681 was propagated in mosquito‐larva cells (C6/36) using HyClone™ Leibivitz L‐15 medium (GE Healthcare). The titer for virus viability and serotype specificity was confirmed by plaque titration assay and nested RT‐PCR, respectively (Baer & Kehn‐Hall, 2014; Lanciotti, Calisher, Gubler, Chang, & Vorndam, 1992). Throughout this experiment, virus infection was performed at a multiplicity of infection (MOI) of 1 PFU/cell. The virus was added to the host cells and incubated at 37°C in 5% CO_2_ in a humidified incubator for 90 min, to allow host‐cell infection. Then, the cells were washed twice with 1× PBS to remove excess virus and serum‐free medium was added for further incubation. The cells were harvested at specific experiment time‐points according to the viral life cycle processes, consisting of adsorption, viral fusion, protein translation/genome replication, viral assembly, viral maturation, and viral release (Mukhopadhyay, Kuhn, & Rossmann, 2005). We harvested the cells at 1, 12, and 36 hr postinfection, as adjusted from previously described studies (Barth, 1992; Mosso, Galvan‐Mendoza, Ludert, & del Angel, 2008; Shrivastava, Sripada, Kaur, Shah, & Cecilia, 2011; Thepparit, Phoolcharoen, Suksanpaisan, & Smith, 2004).

### 2.3 In silico analysis

To preliminarily investigate the possibility of NMT interaction with DENV protein, the computational bioinformatics tool ExPASy ScanProsite (https://prosite.expasy.org/scanprosite/) was used to screen for the *N*‐myristoylation prediction site (Prosite PS0008) from complete whole protein sequences of DENV Serotypes 1–4 according to the consensus sequence G‐{EDRKHPFYW}‐x(2)‐[STAGCN]‐{P}[Gisthe*N*‐myristoylationsite] (Grand, 1989; Hulo et al., 2006; Towler, Gordon, Adams, & Glaser, 1988). The DENV strains were selected from those available on the NCBI database (http://www.ncbi.nlm.nih.gov/protein/) by random sampling from different countries of origin, gathering 10 complete protein sequences from each serotype (Appendix Table A1). The data were analyzed utilizing a percentage calculation for each *N*‐myristoylation prediction site within the DENV genome, and a heatmap was generated using RStudio version 1.1.423. To confirm our preliminary results, a protein–protein docking analysis was performed to screen the overall interaction and compare the two most possible candidate DENV proteins, using protein x‐ray crystallization structures available on Protein Data Bank (PDB) (http://www.rcsb.org/). The candidate proteins were DENV Type 2 envelope protein (PDB ID: 1OAN) and full‐length NS5 protein of DENV Type 3 (PDB ID: 5CCV). Analysis was performed using the ZDOCK online server (http://zdock.umassmed.edu) (Pierce et al., 2014; Vakser, 2014) with human myristoyl‐CoA:protein NMT (PDB ID: 1RXT) and BIOVIA Discovery Studio Visualizer 2.5.

### 2.4 Far‐western blot analysis

To confirm the best single candidate protein result from the molecular docking investigation, we performed a far‐western blot analysis (Rudtanatip, Withyachumnarnkul, & Wongprasert, 2015) with recombinant DENV Type 2 envelope protein (Abcam, Cambridge, UK) and recombinant human NMT protein (Abnova, Taipei, Taiwan), and a positive control. The samples were mixed with 10× sample loading buffer, incubated at 100°C for 5 min, and separated on a sodium dodecyl sulfate polyacrylamide (SDS‐PAGE) gel by electrophoresis for 90 min at 20 mA, 202 V. Then, the proteins were transferred to a polyvinylidene difluoride (PVDF) membrane as a prey protein by semi‐wet blotting at 25 V for 4 hr. Following protein transfer, a bait protein of 5 μg/ml human NMT recombinant protein in PBS was allowed to react with the membrane proteins overnight at 4°C. The binding of nonspecific proteins was then blocked using 4% skim milk in PBS with Tween 20 (PBST) for 2 hr at room temperature and washed three times for 10 min in PBST. After blocking, an NMT1 antibody (Santa Cruz Biotechnology, Dallas, TX) was used as the primary antibody and goat anti‐mouse IgG H&L (HRP) (Abcam) as the secondary antibody. To clarify false‐positive results using the capsid protein, the negative interaction from in silico analysis was used to confirm the positive results of envelope protein. However, conventional western blot needed to be performed as a negative experiment to confirm the far‐western blot, the NMT, and recombinant DENV Type 2 envelope protein, without the bait protein detected by NMT1 antibody as a primary antibody and goat anti‐mouse IgG H&L (HRP) as a secondary antibody. All antigen‐antibody complexes in the western blot were visualized by chemiluminescence detection via a CCD camera (WesternBright™ Quantum™; Advansta, Menlo Park, CA).

### 2.5 Pull‐down assay

The NMT recombinant protein (Abnova) and lysate protein from DENV‐2 strain 16681 infected the induced dendritic cells (iDCs) cell for 48 hr using pull‐down lysis buffer. This experiment was performed with a Pierce™ GST Protein Interaction Pull‐Down Kit (Thermo Scientific, MA). Briefly, as a bait protein the rNMT was immobilized with agarose beads to allow the protein to bind with the affinity ligand for 30 min at 4°C. Then it was washed five times and the DENV‐infected cell lysate was allowed to bind with the bait protein overnight at 4°C. Then, the product was washed five times to remove nonspecific proteins and elute the interaction protein using Glutathione elution buffer; western blot analysis was performed to detect protein using antibody specific for NMT (Santa Cruz Biotechnology) and E glycoprotein (Abcam).

### 2.6 Gene expression by qRT‐PCR

Total RNA was extracted from the dendritic cells infected with DENV‐2 (16681) with MOI 0.1, 1, 10, and the cells harvested at 1, 12, and 36 hr postinfection using a RNeasy^®^ Mini Kit (Qiagen). Viral RNA was extracted from both dendritic cells and supernatant using a QIAamp^®^ Viral RNA Mini Kit (Qiagen) according to the manufacturer's recommendations. The gene expression of human *NMT‐1* was studied using the specific primers NMT‐1 Forward (5′CCGCAGATGATGGAAGGGAA3′) and NMT1 Reverse (5′CCTCTCTGCTGGCAAAGAGTTCA3′) (Takamune, Hamada, Misumi, & Shoji, 2002). The human β‐actin housekeeping gene was used for normalization with the specific primers β‐actin Forward (5′AGAGCTACGAGCTGCCTGAC3′) and β‐actin Reverse 5′AGCACTGTGTTGGCGTACAG3′ (Song & Zuo, 2014). qPCR was performed according to the manufacturer's protocol, using a KAPA SYBR^®^ FAST One‐Step qRT‐PCR kit (Kapa Biosystems) and a CFX96 Touch™ Real‐Time PCR Detection System (Bio‐Rad). To confirm DENV Serotype 2 infection, nested RT‐PCR was performed in all conditions of infection using dengue serotype‐specific primers D1 (5′TCAATATGCTGAAACGCGCGAGAAACCG 3′), D2 (5′TTGCACCAACAGTCAATGTCTTCAGGTTC3′), and TS2 (5′CGCCACAAGGGCCATGAACAG3′). Nested RT‐PCR was performed using a SuperScript™ III One‐Step RT‐PCR System with Platinum™ *Taq* high fidelity DNA Polymerase (Invitrogen) on a T100™ Thermal Cycler (Bio‐Rad). Gene expression was analyzed using the 2(‐Delta Delta C(T)) method (Livak & Schmittgen, 2001) by the expression of the *NMT1* gene with the uninfected control and normalization with β‐actin, the housekeeping gene.

### 2.7 Gene silencing and viral replication assay in NMT1‐knockdown cell

The antisense 2′‐deoxy‐2′‐fluoroarabino nucleic acid oligonucleotides (FANA oligos) of NMT1 and a negative control that was used to perform the gene silencing experiment were designed and synthesized by AUM BioTech and prepared for transfection into the iDCs via gymnotic delivery (Fazil et al., 2016; Souleimanian et al., 2012). The iDCs were seeded at 2.5 × 10^5^ cells/well in a 24‐well plate containing a complete growth medium on the day before treatment with the FANA oligos. The 2.5 μM concentrations of FANA oligo both for NMT1 and negative control were treated directly with the cells mixed with complete medium, to continue cell incubation in humidified incubators. The cells were harvested at 24, 48, and 72 hr for protein extraction and western blot analysis according to the manufacturer's recommendations. Trypan blue staining was performed to determine the effect of silencing on the cells. After the NMT1 knockdown, the DENV was infected to the silencing‐treated iDC and negative silencing with MOI 1. The cellular supernatant was collected 72 hr postinfection for use in the plaque titration assay to determine the productivity of the viable virus.

### 2.8 Western blot analysis

We performed western blots to confirm that the negative experiment of the far‐western blot analysis was successful, and that iDC silencing had been achieved after FANA antisense oligonucleotide treatment. The iDC supernatant was lysed with radioimmunoprecipitation assay buffer to obtain the total protein. The protein concentration was determined by lyophilization and protein quantification using a Pierce™ BCA Protein Assay Kit (Thermo Scientific) to obtain 10 μg of protein. The protein sample was mixed with 10× sample loading buffer, incubated at 100°C for 5 min, and separated by SDS‐PAGE electrophoresis for 90 min at 20 mA, 202 V. Then, the protein was transferred to a PVDF membrane by semi‐wet blotting for 4 hr at 25 V, and the binding of nonspecific proteins was blocked by overnight incubation in 4% skim milk at 4°C. To verify that the iDC‐treated FANA allele‐specific oligonucleotides could silence the *NMT‐1* gene, NMT1 antibody and glyceraldehyde‐3‐phosphate dehydrogenase (GAPDH) (loading control) were used as the primary antibody and goat anti‐mouse IgG H&L (HRP) (Abcam) as the secondary antibody. To confirm the production of DENV envelope protein after virus infection of the knocked‐down cells, anti‐Flavivirus E‐glycoprotein antibody (Abcam) was used as the primary antibody and goat anti‐mouse IgG H&L (HRP) (Abcam) as the secondary antibody. A stripping buffer for antibody restaining was used before the detection of the envelope protein and loading control. The stripping buffer contained 15 g glycine, 1 g sodium dodecyl sulfate, and 10 ml Tween^®^ 20 in 800 ml of distilled water, adjusted pH to 2.2 and a final volume of 1 L. All western blot antigen‐antibody complexes were visualized by chemiluminescence using a WesternBright™ Quantum™ CCD camera.

### 2.9 Plaque titration assay

The supernatant containing the virus was diluted from a 1:10 dilution to fourfold dilutions which were used to infect a monolayer of LLC‐MK2 host cells in 24‐well plates. Following 90 min incubation at 37°C with 5% CO_2_, excess virus was discarded and a carboxymethylcellulose (CMC) substrate was added for further incubation for 7 days, depending on the observation of cytopathic effects under an inverted microscope. The CMC was removed and 3.7% paraformaldehyde was added for 20 min to fix the cells. Then, the cells were stained with 0.1% crystal violet in 20% ethanol. The plate was washed twice with PBS and tap water until the plaques were clearly visible and were counted as plaque‐forming units (PFUs) (Hamel et al., 2015).

### 2.10 Immunogold staining by transmission electron microscope

Immunogold staining was used to confirm the silencing of NMT1 related with envelope protein synthesis, to imply the replication ability of DENV in dendritic cells in the NMT silencing condition. The cell pellet was fixed with 2.5% glutaraldehyde for 1 hr and washed three times with glucose phosphate buffer before processing and gold staining with the desired antibody, NMT1 antibody (Santa Cruz Biotechnology) and anti‐Flavivirus E‐glycoprotein antibody (Abcam). The sample was processed for viewing under a model HT7700 transmission electron microscope (TEM) (HITACHI Ltd., Japan).

### 2.11 Statistical analysis

All experiments were performed in triplicate and the data were presented as mean ± *SD*. The two‐tailed Student's *t* test was used to assess the point of statistical significance between the test groups (*p *< 0.05), indicated by an asterisk (*).

## 3 RESULTS

### 3.1 E protein is the most frequently predicted *N*‐myristoylation site: in silico analysis

**Figure 1:**
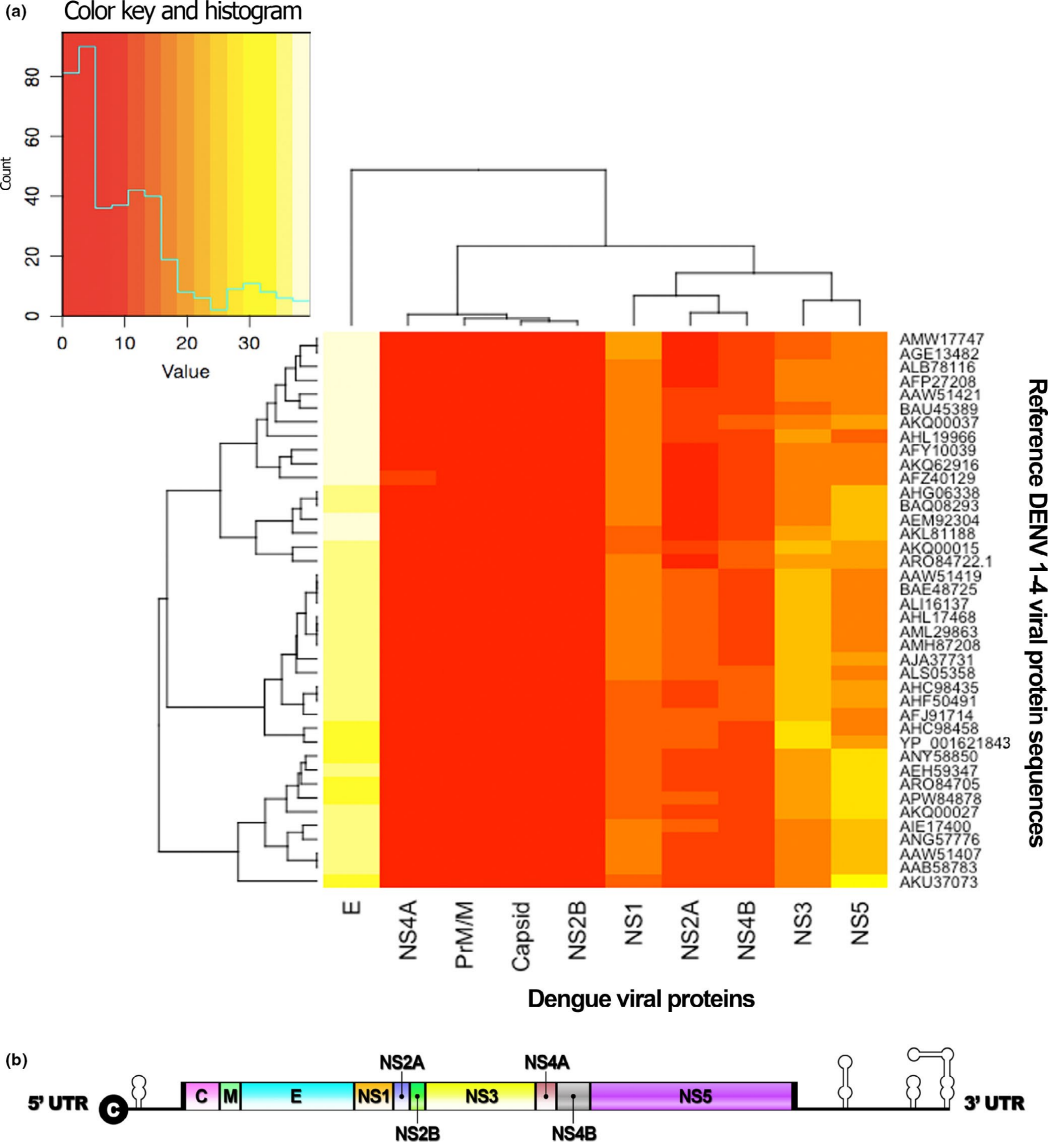
Heatmap shows the dendrogram arrangement of *N*‐myristoylation site prediction catalyzed by *N*‐myristoyltransferase in each dengue viral protein of dengue virus (DENV) Serotypes 1–4 (Appendix Table A1). The envelope protein was highly shared at the predicted *N*‐myristoylation site, followed by NS5, NS3, and NS1, whereas NS4A showed the lowest predicted site of *N*‐myristoylation (a). The normal arrangement of the DENV viral protein is shown in part b

The dendrogram arrangement of the heatmap from the protein sequences of several strains of DENV Serotypes 1–4, which were available on the NCBI database, was analyzed with ExPasy ScanProsite to determine the *N*‐myristoylation site, catalyzed by NMT. The heatmap and dendrogram results revealed that most *N*‐myristoylation prediction sites in complete DENV viral protein sequences are in the DENV‐E protein, followed by NS5, NS3, NS1, NS4B, NS2A, PrM/M, NS2A, NS4B, and C proteins, respectively (Figure 1a). The DENV protein arrangement sequence is indicated in Figure 1b. The two most likely DENV viral protein candidates, DENV‐E and NS5, were to be confirmed using protein–protein interaction docking methods in further experiments.

### 3.2 The E and NS5 DENV proteins bind with hNMT1

**Figure 2:**
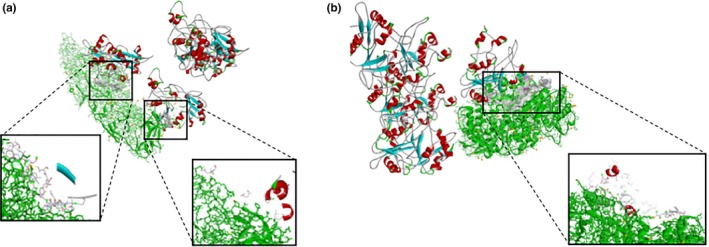
Protein–protein interaction of (a) hNMT: 1RXT (tetramer ribbon protein complex) and dengue virus (DENV) 2 envelope protein: 1OAN (green) with the binding pocket (white) and (b) hNMT: 1RXT (tetramer ribbon protein complex) and DENV NS5 protein: 5CCV (green) with the binding pocket (white). The envelope protein showed two interaction sites, while NS5 showed only one interaction site

We performed a protein–protein interaction analysis on the two predicated DENV candidate proteins, DENV‐E and NS5, determined from in silico analysis. The results indicated that both proteins interacted with hNMT1. The tetramer structure of hNMT1 (PDB: 1RXT) protein had two binding pockets on DENV‐E (PDB: 1OAN) protein (Figure 2a), while DENV NS5 (PDB: 5CCV) protein had one binding pocket for the tetramer of hNMT1 protein (Figure 2b). Thus, we chose the most interactive protein, DENV‐E, for further experimentation.

**Figure 3:**
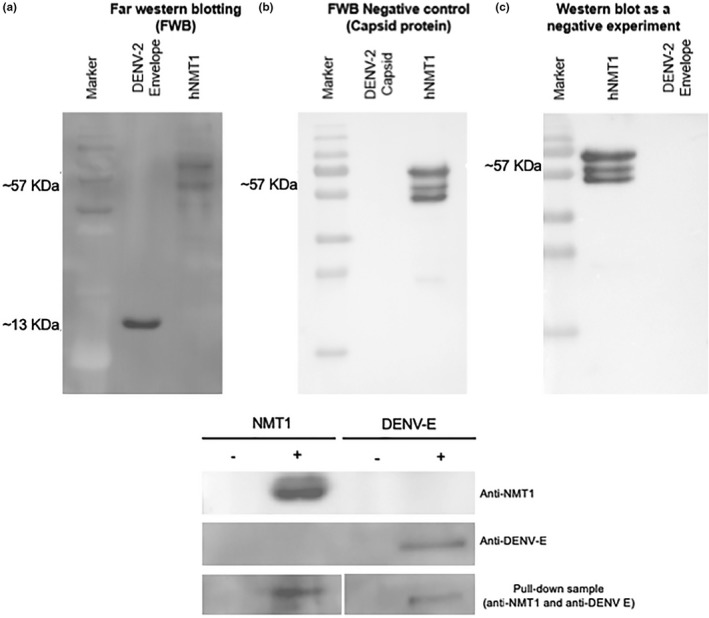
Far‐western Blot analysis of the dengue virus (DENV) E protein with the hNMT protein showed (a) rhNMT protein (bait) strongly interact with rDENV E protein (prey) in the first lane and positive control (*N*‐myristoyltransferase [NMT]), without bait protein, in the second lane; (b) The negative experiment was performed with capsid protein as a prey protein showed no interaction in the first lane; (c) the negative experiment by conventional western blotting presented only hNMT1 interaction in the first lane without interaction in the second lane; (d) the pull‐down assay showed the interaction of E and NMT protein; the first membrane showed the bait control (rNMT1); the second membrane showed the prey protein only, focuses E protein; the third membrane showed the pull‐down sample loading with both bait and prey, resulting from the interaction of NMT1 and E protein

Far‐western blot analysis was used to confirm the protein–protein interaction of the best candidate protein; DENV‐E was used with hNMT1 protein. The results of the experiment showed that hNMT1 bound strongly to DENV‐E protein to form a complex of approximately 13 kDa, and hNMT1 protein formed a band at 57 kDa when used as a positive control (Figure 3a) in our experiment that used hNMT1 as the bait protein and hNMT1 antibody as the primary antibody. DENV capsid protein was used as a negative interaction to exclude false‐positive results. The results revealed no interaction band in the capsid protein lane as a prey protein when using NMT‐1 as a bait protein (Figure 3b). Figure 3c shows a negative experiment by conventional western blotting that was performed without the bait protein. No reaction with the hNMT1 antibody occurred, as can be seen by the absence of a band in the DENV‐E protein lane. The pull‐down assay was used to ensure the protein–protein interaction by using NMT1 recombinant protein with DENV‐2 16681 infected iDC lysate. The result showed the interaction of NMT1 and E protein (Figure 3d) loading control with positive (NMT1 as bait control and E protein as prey control).

### 3.3 NMT1 gene expression related to the life cycle of DENV

**Figure 4:**
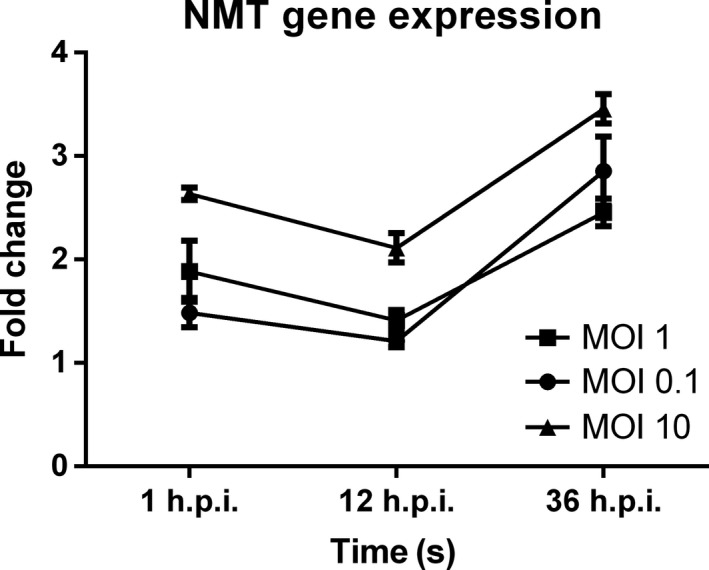
The triplicate gene expression experiment of hNMT1 in dengue virus (DENV) infected induced dendritic cells used qRT‐PCR relative and normalized with uninfected cells and beta actin as a housekeeping gene. The significant upregulation of hNMT‐1 in the DENV‐infected cells showed in 1 h.p.i. with slight downregulation in 12 h.p.i. and marked upregulation in 36 h.p.i. associated with the membrane involved. The difference in multiplicity of infection (MOI) (0.1, 1, 10) with the level of NMT1 expression was MOI‐dependent

*NMT1* gene expression was analyzed by qRT‐PCR to determine whether DENV infection of iDCs induced the expression of the *NMT1* gene. This was investigated at different time‐points of infection, 1, 12, and 36 hr, according to the DENV life cycle in human target cells. Our results (Figure 4) revealed that at 1 hr postinfection, *NMT1* gene expression was significantly upregulated, while at 6 hr postinfection, slight downregulation was observed, reaching a significantly different expression level at 36 hr postinfection, where strongly significant upregulation occurred.

### 3.4 Decreased DENV viral titer in hNMT1 silencing iDC

**Figure 5:**
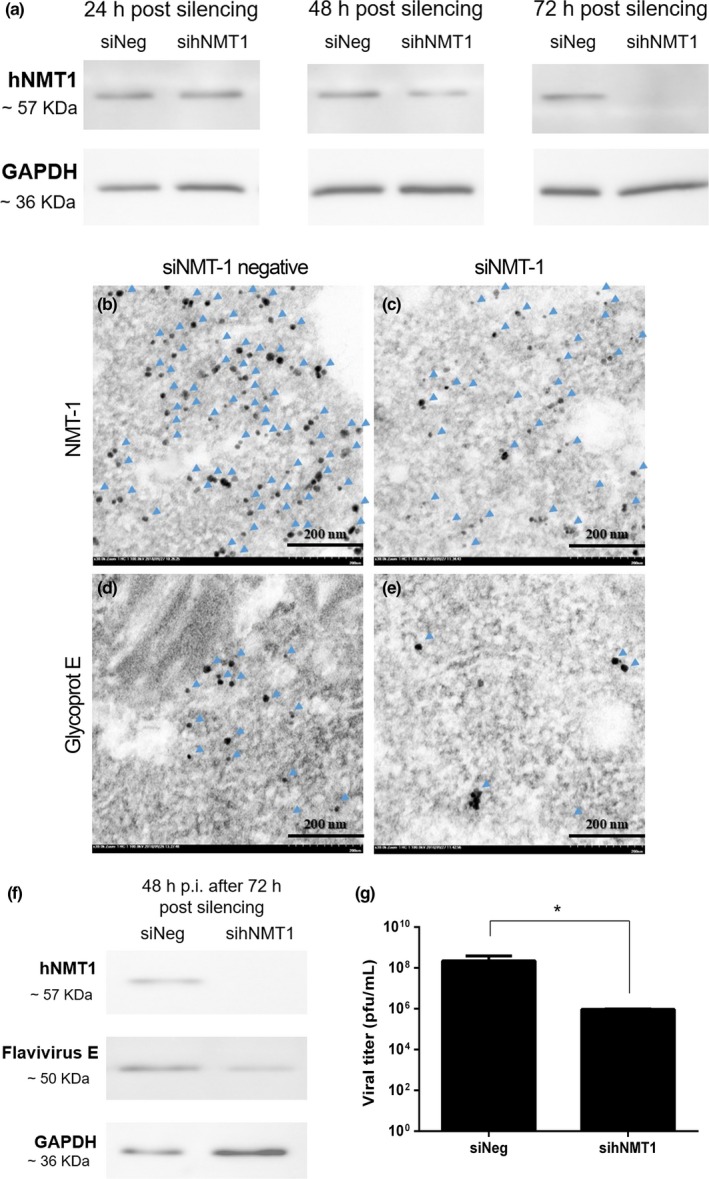
The western blotting of (a) induced dendritic cell (iDC) treated with 2′‐deoxy‐2′‐fluoroarabino nucleic acid oligos for silencing, shown the beginning of silence at 48 h.p.i. and complete silencing at 72 h.p.i. with the GAPDH loading control. The immunogold detection of NMT1 and dengue virus (DENV) envelope protein in siNMT. The present of highly density of NMT1 in negative silencing (b) compare with siNMT1 condition (c). While, the high number of envelope protein showed in negative silencing (d) rather than silencing condition (e). (f) The virus replication assay of 48 h.p.i. by DENV infected the iDC after 72 hr of silencing, examined by western blotting, showed the partially downregulation of flavivirus E protein in siNMT. (g) triplication of plaque titration assay to investigate the viral titer, showed the significantly decreasing of viral production in siNMT cell

The FANA oligos were introduced to the first iDC passage together with complete growth medium. The investigation time‐points were the depletion of hNMT1 at 24, 48, and 72 hr, according to the manufacturer's recommendations. The results showed that hNMT1 started to deplete 48 hr post‐silencing treatment, with obvious depletion at 72 hr (Figure 5a) without evidence of a negative silencing effect or effect on the viability and attachment ability of the silenced cells, as determined by trypan blue staining and observation, respectively (data not shown). Thus, we chose the cells for the viral replication examination assay at 72 hr post‐silencing treatment. The iDC at 72 hr posttreatment with FANA oligos was infected with DENV2‐16681 at a MOI of 1 plaque‐forming unit (PFU)/cell. Following 48 hr incubation at 37°C in a humidified incubator with 5% CO_**2**_, the supernatant was collected to investigate virus viability and production by plaque titration assay. Immunogold staining showed a high density of NMT‐1 in the absence of NMT‐1 silencing (Figure 5b), related to the high amount of DENV envelope protein presented (Figure 5d). The NMT‐1 silencing condition showed low amounts of NMT‐1 than without the NMT‐1 silencing condition (Figure 5c), which also showed a lower amount of DENV envelope protein (Figure 5e). The protein was extracted from the cells to perform western blot analysis. The results revealed the DENV‐E protein downregulation, not complete inhibition, when compared to negative silencing and the GAPDH loading control Figure 5f). Hence, we determined that the titer of viable virus from the plaque titration assay was significantly downregulated in the iDC silencing treatment when compared to negative silencing (Figure 5g).

## 4 DISCUSSION

Numerous studies have identified significant viral‐host interactions in several kinds of viruses, and DENV‐host interaction has been established as necessary for the viral replication cycle, antiviral response, and as a possible approach for antiviral targets (Acosta, Kumar, & Bartenschlager, 2014). In this study, we examined the role and efficiency of hNMT1 during DENV replication. First, we performed in silico analysis to further test the DENV‐host interactions related to the replication cycle in dendritic cells, the primary sentinel human target cell and the primary target for DENV replication and mediated immunity (Marovich et al., 2001; Schmid, Diamond, & Harris, 2014). We included DENV Serotype 2 strain 16681 in the in silico analysis (Figure 2), which showed that DENV‐2 had a high abundance of predicted *N*‐myristoylation sites and that DENV‐2 strains were associated with severe dengue cases and most frequently found in worldwide epidemics (Nunes et al., 2016; Wei & Li, 2017). DENV strain 16681 was isolated from a patient with a severe case of dengue fever and has been widely used to study DENV pathogenesis (Okamoto et al., 2012). The in silico results showed that *N*‐myristoylation sites were abundant in DENV serotypes, correlating with the findings of Maurer‐Stroh and Eisenhaber (2014), who identified myristoylation sites in four serotypes of DENV using the NS5 protein sequence as a predictor (Maurer‐Stroh & Eisenhaber, 2014). However, our study used complete DENV protein sequences, and determined that the most frequent myristoylation sites were DENV‐E, NS5, and NS3, respectively. This is also supported by Ruhul Amin et al. (2010), who discovered abundant myristoylation sites on the DENV‐E protein of four DENV serotypes (Ruhul Amin , Mahbub, Sikder, & Karim,

2010), while two previous in silico studies suggested that the myristoylation process in the DENV viral protein might be involved with membrane‐associated viral entry to the host cell and other pathogenic mechanisms. Our finding of abundant myristoylation sites on DENV NS3 concurs with Heaton et al. (2010), who demonstrated that DENV NS3 associated with fatty acid synthase at the site of DENV replication and was also involved in fatty acid biosynthesis (Heaton et al., 2010; Salazar, del Angel, Lanz‐Mendoza, Ludert, & Pando‐Robles, 2014). This is compatible with the theory of the myristoylation process (Resh, 1999).

We used the best two candidate proteins from the in silico analysis, E and NS5 proteins to perform the protein–protein interaction analysis with hNMT, aimed to screening the overall interaction to provide the information platform for the experimental study. Our results found more interaction pockets in the DENV‐E protein than in the NS5 protein, correlating with the in silico result. Thus, we used the E protein for far‐western blotting, and we detected DENV‐E protein strongly bound to hNMT protein, confirming and excluding false‐positive results using the capsid protein as a negative interaction experiment. Moreover, a pull‐down assay was performed to confirm the interaction of hNMT and DENV‐E protein. The result showed the interaction of these proteins, with a negative control. These experimental results concur with the in silico analysis.

**Figure 6:**
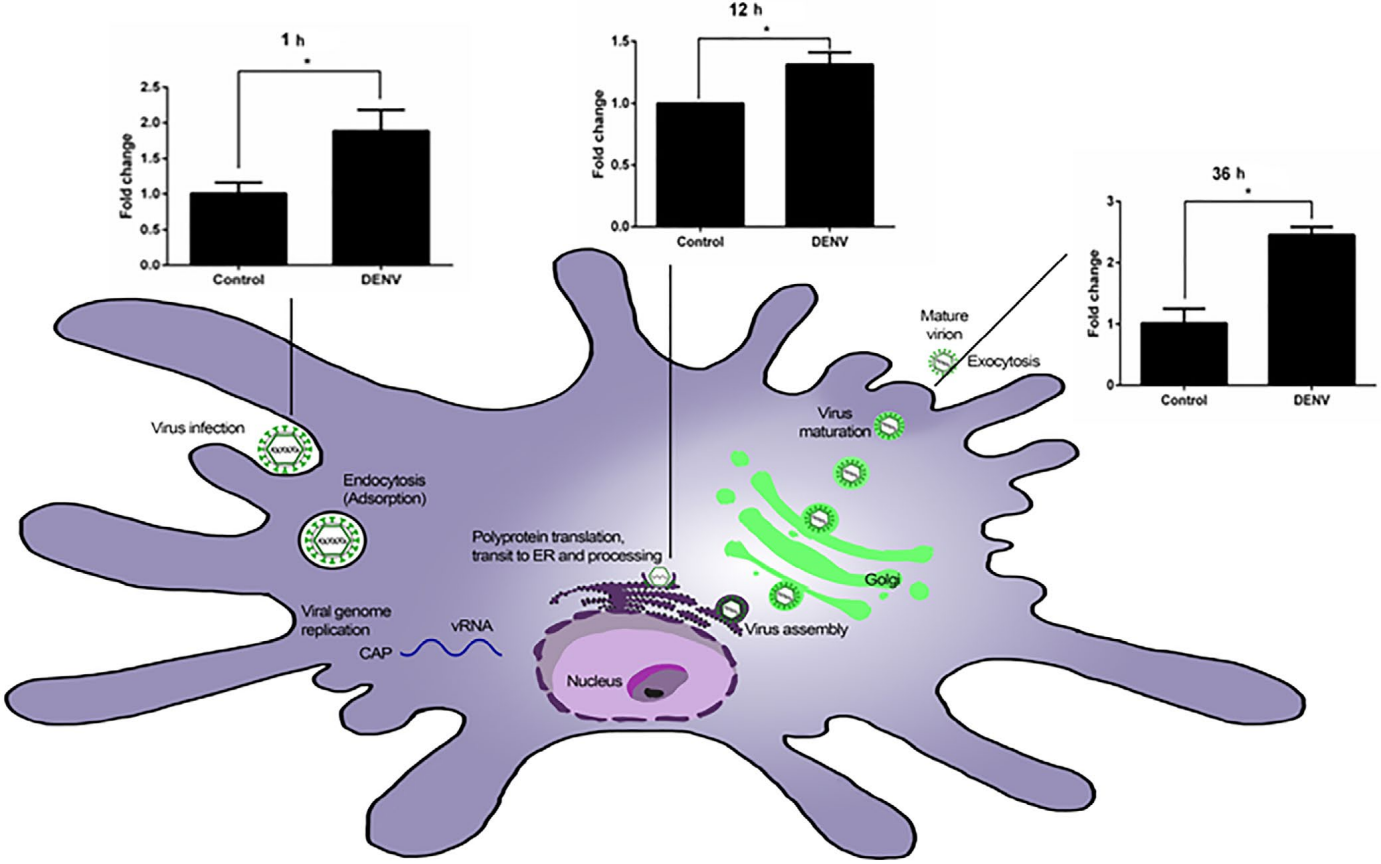
Summary of NMT1 gene expression related with the process of the dengue virus life cycle. Upregulation of the *NMT1* gene in 1, 12, 36 h.p.i. was related to the membrane (endocytosis, protein translation at the ER membrane, and exocytosis), analyzed with the predicted dengue virus replication time

We used the time‐points for DENV replication in target cells from previous studies (Barth, 1992; Mosso, Galvan‐Mendoza, Ludert, & del Angel, 2008; Shrivastava , Sripada, Kaur, Shah, & Cecilia,

2011; Thepparit et al., 2004) to examine *NMT1* gene expression in human dendritic cells at different time‐points postinfection. The results revealed the significant upregulation of NMT1 at 1, 12, and 36 hr, which might represent the time period of DENV infection, and which suggests NMT‐related membrane‐associated viral entry (Abou‐Jaoude, Molina, Maurel, & Sureau, 2007; Maurer‐Stroh & Eisenhaber, 2014; Thaa et al., 2009), transcription and endoplasmic reticulum involvement (Moriya et al., 2013), and viral exocytosis (Figure 6).

To verify the precise role of NMT in DENV infection, we knocked‐down hNMT1 in iDCs to determine DENV replication. Our results indicated DENV‐E protein downregulation when compared with the control, as detected by immunogold TEM and western blotting, the viral viability titer and immunogold staining were significantly decreased, without complete inhibition, in the hNMT1 knockdown iDCs detected by plaque titration assay. It is important to note that hNMT1 might only be one of many host factors that facilitate the replication of DENV and is also involved in membrane‐associated viral entry into host cells.

**Figure 7:**
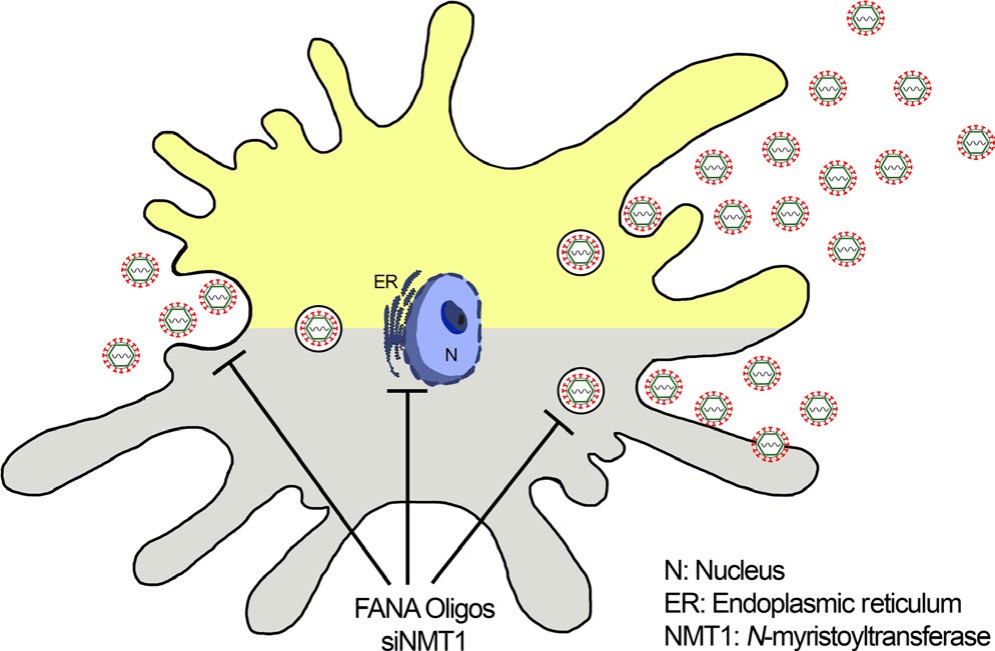
Summary conclusion diagram for this study. The dengue virus (DENV) infected dendritic cell diagram shows DENV replication ability. The upper part shows normal condition, the DENV viral particles could normally produce viral progeny; the lower part shows the NMT1 depletion condition, the restriction of DENV replication leading to low DENV viral progeny production numbers

In conclusion, the results of this study suggest that NMT1 may act as a host factor that facilitates DENV replication through the interaction of a membrane‐involved viral life cycle, especially adsorption, viral assembly, and exocytosis, as summarized in Figure 7. However, future studies should explore other host and viral factors and further investigate DENV‐host interactions in other target cell types. These experimental data suggested that all dengue viral protein need to be investigated for their interaction and role with NMT proteins, for both bioinformatic and laboratory experiments. In addition, other DENV target cells should be studied to clarify the role of NMT under DENV infection conditions.

## CONFLICT OF INTERESTS

The authors declare they have no competing interests.

## AUTHORS CONTRIBUTION

Conceptualization: Natthanej Luplertlop; Data Curation: Natthanej Luplertlop and San Suwanmanee; Formal Analysis: San Suwanmanee and Natthanej Luplertlop; Funding Acquisition: San Suwanmanee, Natthanej Luplertlop and Dorothée Missé; Investigation: San Suwanmanee, Yuvadee Mahakhunkijcharoen, Sumate Ampawong, Pornsawan Leaungwutiwong, Dorothée Missé and Natthanej Luplertlop; Methodology: San Suwanmanee and Natthanej Luplertlop; Project Administration: Natthanej Luplertlop and San Suwanmanee; Supervision: Natthanej Luplertlop and Dorothée Missé; Validation: Natthanej Luplertlop, San Suwanmanee and Yuvadee Mahakhunkijcharoen; Writing‐Original Draft Preparation: San Suwanmanee and Natthanej Luplertlop; Writing‐Review & Editing: San Suwanmanee, Yuvadee Mahakhunkijcharoen, Sumate Ampawong, Pornsawan Leaungwutiwong, Dorothée Missé and Natthanej Luplertlop.

## ETHICS STATEMENT

Buffy coat was purchased from the National Blood Center, Thai Red Cross Society with ethical exemption (TMEC 16‐043) from the Ethics Committee of the Faculty of Tropical Medicine, Mahidol University, Thailand and permission letter no. NBC 10727/2559.

## Data Availability

All data are provided in full in the results section of this paper.

## APPENDIX 1

### 1.1

**Table A1:** The reference dengue virus (DENV) 1–4 protein sequences in in silico analysis with the *N*‐myristoylation predicted site

Serotype	Accession no.	Strain	Country
1	ARO84722.1	TM245	Malaysia
	AHF50491	HNRG28425	Argentina
	AHL19966	CHN/GuangDong/ZhongShan/Henglan01/2013	China
	AFJ91714	DENV‐1/BOL‐KW010	USA
	AEM92304	49440	Myanmar
	BAQ08293	D1/Hu/Hyogo/NIID188/2014	Japan
	AKQ00015	DENV1 BR/SJRP/509/2012	Brazil
	AKL81188	SGEHI(D1)22176Y13	Singapore
	AHG06338	MKS‐0077	Indonesia
	AHC98435	DENV‐1/NI/BID‐V7677/2012	Nicaragua
2	AAB58783	PDK‐53	Attn. 16681 vaccine
	ANG57776	16681	Mahidol, TH
	ARO84705	TM289	Malaysia
	AIE17400	DENV‐2/Pk/Swat‐02	Pakistan
	AKU37073	ZS01/01	China
	AEH59347	DENV‐2/US/BID‐V5413/2009	USA
	APW84878	Homo sapiens/Haiti‐0078/2014	Haiti
	ANY58850	QML16	Australia
	AAW51407	TB16i	Indonesia
	AKQ00027	BR/SJRP/869/2013	Brazil
3	AML29863	Rajasthan.India/Balotra 87‐s/2013	India
	AJA37731	D83‐144	Thailand
	AHL17468	13GDZDVS30E	China
	BAE48725	D3/Hu/TL018NIID/2005	Timor‐Leste
	AAW51419	TB55i	Indonesia
	AMH87208	Cuba_20_2002	Cuba
	ALS05358	H87	Philippines
	ALI16137	DENV‐3/KBPV‐VR‐30	South Korea
	YP_001621843	D3/H/IMTSSA‐SRI/2000/1266	Sri Lanka
	AHC98458	DENV‐3/NI/BID‐V7699/2011	Nicaragua
4	AGE13482	EHI310A129SY10	Singapore
	AAW51421	SW38i	Indonesia
	AKQ00037	BR/SJRP/850/2013	Brazil
	AMW17747	MRS 6169642904/2014	Haiti
	ALB78116	H241	Philippines
	BAU45389	D4/Hu/India/NIID48/2009	Japan
	AKQ62916	DENV4/CN/GZ29/2010	China
	AFP27208	DSS patient	Cambodia
	AFY10039	WF09/010409‐0001	Wallis and Futuna
	AFZ40129	DENV‐4/IND/793679/1979	India
